# Animal Models in Studying Cerebral Arteriovenous Malformation

**DOI:** 10.1155/2015/178407

**Published:** 2015-11-16

**Authors:** Ming Xu, Hongzhi Xu, Zhiyong Qin

**Affiliations:** ^1^Department of Anesthesiology, Huashan Hospital, Fudan University, Shanghai 200040, China; ^2^Department of Neurosurgery, Huashan Hospital, Fudan University, Shanghai 200040, China

## Abstract

Brain arteriovenous malformation (AVM) is an important cause of hemorrhagic stroke. The etiology is largely unknown and the therapeutics are controversial. A review of AVM-associated animal models may be helpful in order to understand the up-to-date knowledge and promote further research about the disease. We searched PubMed till December 31, 2014, with the term “arteriovenous malformation,” limiting results to animals and English language. Publications that described creations of AVM animal models or investigated AVM-related mechanisms and treatments using these models were reviewed. More than 100 articles fulfilling our inclusion criteria were identified, and from them eight different types of the original models were summarized. The backgrounds and procedures of these models, their applications, and research findings were demonstrated. Animal models are useful in studying the pathogenesis of AVM formation, growth, and rupture, as well as in developing and testing new treatments. Creations of preferable models are expected.

## 1. Introduction

Brain arteriovenous malformations (AVMs) are vascular anomalies where arteries and veins are directly connected through a complex, tangled web of abnormal vessels instead of a normal capillary network. There is usually high flow through the feeding arteries, nidus, and draining veins. AVMs represent a high risk for hemorrhagic stroke, leading to significant neurological morbidity and mortality in relatively young adults [1]. How the pathological and hemodynamic features play a role in AVM rupture is unknown.

The management in the case of sudden bleeding is focused on restoration of vital function and prevention of recurrent hemorrhage, usually with some combination of surgical resection, embolization, and stereotactic radiotherapy. But all of these treatments pose a risk of serious complications, and the optimal treatment needs to be evaluated [2]. For nonruptured AVMs, whether the preventive treatments are beneficial is uncertain, because nonintervention may result in favorable long-term outcome [3].

As considered to be embryonic origin and postnatal development, AVMs are highly dynamic rather than static [4, 5]. Angiogenesis or vascular proliferation occurs in the AVM lesion. Understanding the exact molecular mechanisms of AVM formation and progression is critical for developing novel therapies such as the vascular targeting therapy and the gene therapy.

Animal models are warranted to meet the needs mentioned above. Up to now, several experimental animal models have been developed in studying the AVM-related hemodynamics, pathogenesis, and treatments. Hence, a review was made about the background, the procedure, and the application of these models, and their advantages and disadvantages were briefly analyzed.

The aim of the review was to encourage creating more advantageous AVM models and promote further studies of the disorder.

## 2. Methods

We searched PubMed till December 31, 2014, using the term “arteriovenous malformation,” limiting results to animals and English language.

Two investigators read the titles and abstracts of the publications to find out the possibly relevant ones that described creations of AVM animal models or investigated AVM-related mechanisms and treatments using these models. The articles describing the creation of the dural arteriovenous fistula models or AVM lesions in other organs were excluded. Full texts of the selected articles were obtained, and those fulfilling our inclusion criteria were identified and finally summarized.

The emphases of the review were on the background, the procedure, and the application of each particular model. The chosen animals, the advantage, and the disadvantage of each model were also briefly discussed.

## 3. Results

From the result of total 911 publications found according to the search term, we picked up more than 100 articles, by the inclusion criteria of either describing the creation of original or modified animal models or adopting these models to make experimental researches.

The animal models in the study of AVMs were diverse in accordance with research purpose, ranging from those based on the changes of the cerebrovascular circulation to those based on gene manipulation techniques. Eight different types of the original models were summarized and their highlights were shown in Tables 1 and 2.

### 3.1. The Carotid-Jugular Fistula

**Figure 1:**
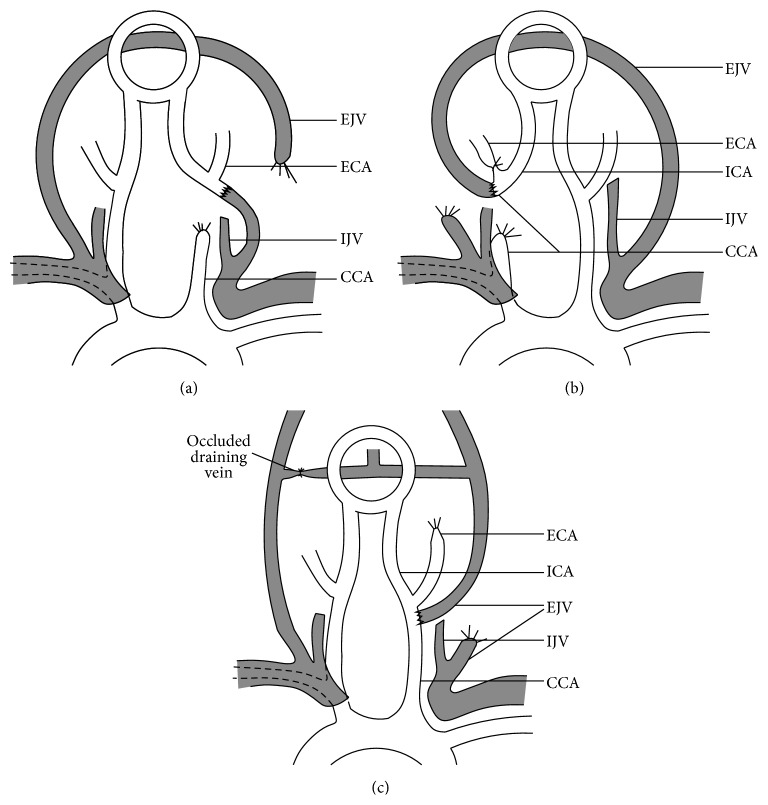
Animal models with carotid-jugular fistulae. (a) Spetzler's model, (b) Morgan's model, and (c) Hai's model. CCA: common carotid artery; ICA: internal carotid artery; ECA: external carotid artery; EJV: external jugular vein; IJV: internal jugular vein.

To explain a phenomenon that brain tissue surrounding the AVM lesion is subject to swelling and hemorrhage immediately following surgical excision of the lesion, Spetzler et al. firstly suggested that the chronic ischemic brain tissue near high flow AVMs might experience a loss of vascular autoregulatory capacity, the theory of normal perfusion pressure breakthrough (NPPB), by using carotid-jugular fistula (CJF) model in cats [6]. This model was created by means of an anastomosis between the rostral end of the common carotid artery (CCA) and the caudal end of the external jugular vein (EJV) together with the ligation of the remaining vessel stumps, so that noninfarction cerebral hypoperfusion was achieved by draining the blood from the circle of Willis retrogradely through the anastomosis (Figure 1(a)). After 6 weeks, only the animals with marked dilatation of the fistula vessels exhibited diminished cerebrovascular autoregulation with both open and closed fistulae, indicating the detrimental effect of high flow through AVMs on surrounding tissues. The other investigators reevaluated this cat model but found that the cerebrovascular hemodynamic changes were actually minimal and transient by the CJF formation and systematic blood pressure interference, and CO_2_ reactivity in the closed fistula was preserved. This model was probably not enough to clarify the mechanisms of the NPPB phenomenon [36–38].

Therefore, a modified CJF model in rats was introduced by Morgan and colleagues. They made an end-to-end anastomosis of both rostral ends of the CCA and the EJV (the internal jugular vein in rats is hypoplastic, and the cerebral venous blood drains mainly to the EJV) on the right side and ligated the caudal ends of both vessels and the ipsilateral external carotid artery (ECA), creating a functional arteriovenous fistula between the circle of Willis and the right lateral sinus (Figure 1(b)). After a period of 8 to 12 weeks, the presence of CJF significantly reduced the cerebral blood flow (CBF) on the fistula side compared to the baseline. Fistula closure significantly elevated CBF, causing the blood-brain barrier (BBB) breakdown under induced hypertension, but not under a normal pressure [7, 39, 40]. Further studies verified that the histopathological change of the cerebral capillaries was the structural basic of the NPPB phenomenon [41]. Interestingly, the CO_2_ reactivity of cerebral vessels remained intact throughout the experiment. The research group recommended the avoidance of intraoperative hyperventilation and postoperative hypertension for the removal of AVM lesions. By using this model, a research group tested the hypothesis that intracerebral, extracellular norepinephrine could be the key factor influencing CBF levels [42], and another group evaluated the effect of ionizing radiation on the blood-stolen parenchyma and concluded that the radiotherapy-related damage in the normal or the hypoperfused brain tissues was similar [43].

Besides, “occlusive hyperemia” was also suggested to be related to the brain edema and hemorrhage following the large AVM resection. High blood flow and mass effect of AVM lesions might cause obstruction of the venous outflow and stagnation of arterial inflow in their adjacent parenchyma, with subsequent worsening of the existing hypoperfusion and ischemia in these tissues. Bederson et al. evaluated this presumption in a rat CJF model by a proximal CCA to distal EJV anastomosis with contralateral EJV occlusion [8]. The fistula significantly increased torcular pressure and decreased systematic pressure, and the venous occlusion for one week caused venous infarction, subarachnoid hemorrhage, and severe brain edema. Based on this, Hai et al. developed a more moderate model of chronic cerebral hypoperfusion combined with draining vein hypertension, by an end-to-side anastomosis between the EJV and the CCA on the right side with ligations of bilateral ECAs and the left vein draining the transvers sinus (Figure 1(c)). After 90 days, occlusion of CJF led to the NPPB phenomenon, which was further demonstrated to share similar pathological mechanisms with acute ischemia reperfusion injury such as infiltration of inflammatory cells and activation of oxygen free radicals [9, 44]. Hemodilution with high-concentration human serum albumin has a certain pretreatment effect on this brain injury [45]. Kojima et al. created very similar rat CJF models with not only the drop in perfusion pressure but also the impaired draining venous outflow [46].

Rats were mostly chosen as the model animal probably because they are economic and accessible in spite of their anatomical differences related to humans. CJF models were also tried in monkeys; however, they were hard to handle, expensive to create, and also with intricate ethical concerns [10].

### 3.2. The Intracranial Arteriovenous Fistula

Carotid-jugular fistulae resulted in the hemodynamic changes in whole brain or predominantly the hemisphere in the fistula side, but not in the regional parenchyma. A dog model with local cerebral hypoperfusion was tried using an intracranial arteriovenous fistula [11]. The dog was chosen not only because its brain was large enough for operation, but also because the physiology and hemodynamic situation were comparable between the dog and human brains. After craniotomy, a fistula was created by a femoral venous graft with end-to-side anastomosis both to the cortical branch of the middle cerebral artery (MCA) and to the superior sagittal sinus (SSS). Shunt opening markedly decreased regional CBF (rCBF) in the MCA territory, but not in other areas. Shunt reocclusion caused rCBF to rebound and return to the preopening value within 15 minutes. Regional CO_2_ reactivity decreased significantly at shunt opening. The regional hemodynamic changes in this animal model simulated a real condition of brain tissues surrounding human AVMs. However, this was an acute model and the procedure was a bit complicated.

Both extracranial and intracranial arteriovenous fistula models lacked a real AVM nidus, these models were focusing on the hemodynamic and pathophysiological changes of AVM adjacent parenchyma, but not the AVM lesion itself.

### 3.3. The Rete Mirabile as the AVM Nidus

**Figure 2:**
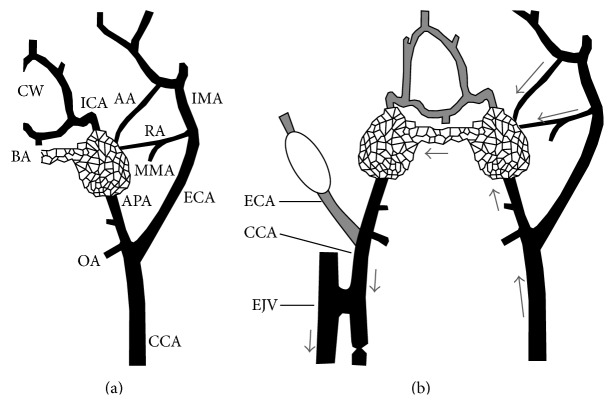
Anatomic basis and features of the swine AVM model. (a) Schematic representation of the normal left carotid arterial anatomy of the swine. The carotid rete mirabile is situated at the termination of the APA. ICA: internal carotid artery; ECA: external carotid artery; CCA: common carotid artery; IMA: internal maxillary artery; MMA: middle meningeal artery supplying the ramus anastomoticus; RA: ramus anastomoticus; AA: arteria anastomotica; APA: ascending pharyngeal artery; OA: occipital artery; BA: basilar artery; CW: circle of Willis; EJV: external jugular vein. (b) Schematic representation of the AVM model after creation of a right carotid-jugular fistula. Arrows indicate direction of flow, that is, from the left CCA to both retia mirabilia via the three feeding arteries (the left APA, RA, and AA), and retrograde down the right APA toward the right carotid-jugular fistula. Note balloon occlusion of the right ECA.

The carotid rete mirabile (RM) of the swine is a special vascular structure with a tangle of microarteries and arterioles situated at the termination of each ascending pharyngeal artery (APA) as it perforates the cranial base. The two sides of the RM, which are connected with each other across the midline, are also supplied by other small collateral arteries and effuse to form internal carotid arteries ipsilaterally (Figure 2(a)). At the end of 1980s, several authors began to report that the swine RM could be used as the AVM nidus to evaluate different materials for embolization and the single-dose radiation effects, due to their morphological similarities [47–50]. The occlusive effect of the treatments could be evaluated by superselective angiography and histopathological observation. An important distinction between the RM structure and a real AVM nidus is the hemodynamic difference; the former is arterioarterial system, but the latter is an arteriovenous system with a higher pressure gradient between feeding and draining vessels.

To address this shortfall, Chaloupka et al. produced a high flow arteriovenous shunt in the swine RM by inserting a needle through the orbit to create communications between the rete and the surrounding cavernous sinus [12]. Superselective angiography into the APA showed rapid sequential filling of the rete, cavernous sinus, and basilar sinus. However, this model had limitations of obvious eye complications, spontaneous occlusion of the arteriovenous shunt, and being only for short-term investigations.

Massoud et al. developed a distinguished swine AVM model with induced high blood flow across both retia, by surgical formation of a side-to-side arteriovenous fistula between the CCA and the EJV with the ligation of the CCA proximal to the fistula on the right side [13]. The angiography showed a clear demonstration of the feeding arteries (mainly the left APA), the nidus (bilateral retia), and the draining vein (the right APA down to the fistula), very similar to human AVMs (Figure 2(b)). An average blood pressure of the left APA dropped from 77 mmHg to 67 mmHg after model formation, and the right APA pressure dropped further to 46 mmHg. By additional occlusion of the rete branches on the right side, the research group also successfully preserved the same model for follow-up study up to 180 days [51]. In the chronic model, striking transmural changes of nidus vessels were observed, representative of realistic histopathologic features in human AVMs. Both the acute and the chronic models were widely adopted in the study of AVMs [52–58], especially in the aspects of hemodynamic changes, embolization therapy, and radiosurgery.

Based on Massoud's model, modified swine AVM models were introduced. They posed a higher pressure gradient closer to values found in human AVMs, thereby reducing the rate of spontaneous thrombosis in the rete [59, 60].

**Figure 3:**
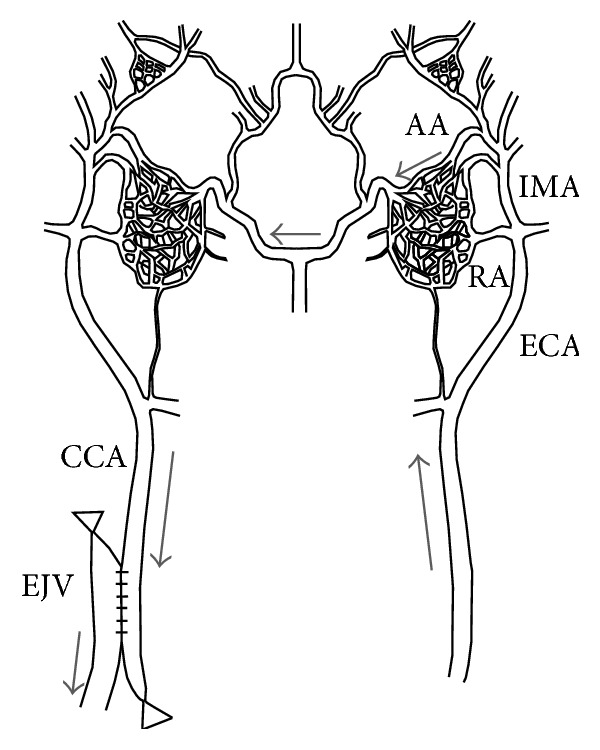
Anatomic basis and features of the sheep AVM model. Arrows indicate direction of flow, that is, from the left side of the carotid artery through both retia mirabilia, retrograde to the right carotid artery and jugular vein following surgical creation of an anastomosis. CCA: common carotid artery; ECA: external carotid artery; IMA: internal maxillary artery; RA: ramus anastomoticus; AA: arteria anastomotica; EJV: external jugular vein.

Besides, in the pig, the natural structure of carotid RM is also seen in the other artiodactyl animals such as the sheep, goat, ox, and cat, but not in the dog, rabbit, and rat. Whether the swine RM models can be duplicated in the other animals was unknown, except for a feasibility study in the sheep [14]. The vascular structure and blood supply of the RM in the sheep (the ascending pharyngeal artery is atrophy) slightly differ from those in the pig. A sheep AVM model was successfully created by a side-to-side surgical anastomosis between the CCA and the EJV with ligations of the vein above and the artery below the anastomosis (Figure 3). An angiographic appearance was demonstrated to simulate human AVMs in all the animal models. Creating the sheep model was rather simple and cost-effective, but it was not routinely adopted in AVM study.

### 3.4. The Extracranial Venous Plexus as the AVM Nidus

**Figure 4:**
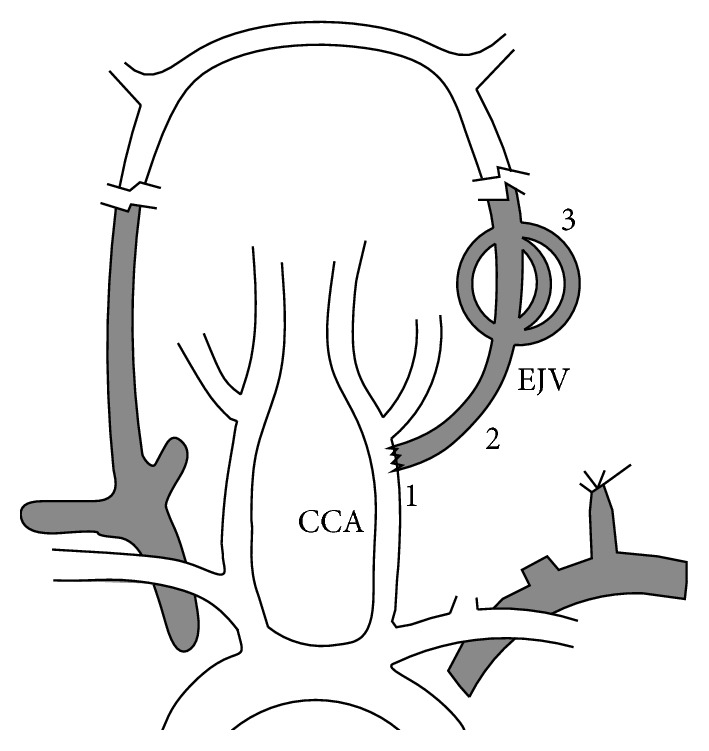
The arteriovenous fistula of the rat arteriovenous malformation model. 1: fistula; 2: arterialized jugular vein; 3: nidus; CCA: common carotid artery; EJV: external jugular vein.

In 2004, Yassari et al. described a rat model with the sham AVM nidus simply by ligating the left EJV at the confluence of the subclavian vein and making an end-to-side anastomosis of the EJV to the CCA [15]. These rats were observed up to 90 days. Angiographic and hemodynamic examinations showed that a high blood flow was diverted across fistula into the EJV (as the feeding artery), through a network of venous branches (as the nidus), then reconnected, and drained to the sigmoid sinus (as the draining vein), presenting a similar feature as in human AVMs (Figure 4). The high flow occurred immediately and kept stable after fistula formation, while the mean pressure in the fistula significantly dropped on day 7 and tended to stabilize by day 21.

Further analysis in this model demonstrated that the nidus vessels underwent morphological changes from normal veins to those similar to immature vessels in human AVMs, including heterogeneously thickened walls, splitting of the elastic lamina, and thickened endothelial layers [61]. Another study found out that the endothelial molecular changes in the nidus occurred, such as increased expression of vascular endothelial growth factor (VEGF), also similar to those observed in human AVMs [62]. These findings supported the theory that vascular changes in AVMs are secondary to increased flow rather than a primary phenotypic abnormality.

The activation of vascular cells in the nidus made it a unique model for studying the occlusive effect of radiosurgery on AVM vessels, because little was known about the molecular mechanisms of radiation mediated vascular obliteration. One study using the model showed that the expression of endothelial adhesion molecules in the nidus cells changed after radiosurgery [63]. Other studies tried to seek strategies to enhance AVM obliteration and reported an improved obliteration rate by induced thrombosis in the nidus with radiosurgery and coadministration of low-dose lipopolysaccharide and soluble tissue factor [64].

### 3.5. The AVM-Like Lesion Derived from Implants

Both the AVM lesions with simulated niduses using the RM and the venous plexus did not actually locate in the cerebral parenchyma. Pietilä et al. developed a novel model with an induced AVM lesion in the dog brain [16]. A vascular bypass was created between the MCA and the SSS by interposing a superficial temporal artery (STA) segment. A muscle graft supplied by a branch of the interposed vessel segment was implanted in the blood-stolen brain area due to the arteriovenous shunt. Postoperative angiography after 6 months demonstrated the feeding artery (the STA segment near the MCA) and the dilated draining vein (the STA segment near the SSS). Between them, AVM-like lesions with newly developed vessels were seen surrounding the muscle implant. The histopathological examination after 8 months demonstrated pronounced gliosis and endothelium/capillaries proliferation in this area. All proliferating vessels had delicate walls and small lumens and lacked differentiation into arterial and venous vessels. These suggested that AVM lesions in the adult brain could develop in the course of time, primarily as a result of angiogenesis, on the condition of cerebral ischemia and/or venous hypertension. The idea of using a pedicle muscle graft as a stimulus for inducing the intracerebral AVM-like lesion was derived from observational and therapeutic studies of Moyamoya disease.

There were some highlight features of this model resembling the appearance of AVMs in human, including thickening and fibrosis of the draining venous wall, new formation of vessels, and vascular proliferation, surrounding brain tissues with signs of ischemia and hemorrhage. Although an exquisite surgical technology was required for producing the animal model, it might help discovering the pathological mechanisms involved in AVM development.

### 3.6. The Xenograft Arteriovenous Fistula

Currently, radiosurgery was a kind of less invasive treatment for AVMs. It took a therapeutic effect by obliterating the AVM nidus, with a low obliterating rate and a latency period up to 2 years. Further understanding of the mechanism of radiosurgery might be helpful to develop advanced pharmacological therapies to improve the occlusive effects based on conventional radiosurgery.

For this purpose, the xenograft arteriovenous fistula model was created, as a segment of main arteries from transgenic mice was interposed between the caudal end of the CCA and the rostral end of the EJV in immune-deficient nude rats [17]. The implanted arterial graft was not a real AVM nidus but shared the AVM hemodynamic features with low resistance and high flow. Mice were chosen as the resource of donor arteries because diverse transgenic mice were available. The small size of mice made homotransplantations difficult, so rats were chosen as the receptor.

In this model, the arteriovenous fistula with radiation pretreatment reproduced distinct radiation arteriopathy as observed in resected human AVM specimens pretreated with radiosurgery. If radiation pretreatment would result in a specific molecular change in the fistula graft, or if the fistula graft from different transgenic mice would have a different response to radiation, this model probably yielded clues to the vascular targeting therapy and the gene therapy. One study had detected that some robust but modified radiation responses occurred in Endoglin and eNOS knockout transgenic arteriovenous fistulae [65].

The model was technically feasible and the overall angiographic patency rate was about 50%. However, there was a time limitation of 4 months for allowing transplanted tissues to retain their phenotypes due to the rejection reaction.

### 3.7. The Rat Cornea with Human AVM Tissues

The surgically resected human AVM lesions were valuable specimens for the histopathological study. When the specimens were transplanted into the corneal micropocket of the rats, they kept alive and growing. The angiogenic activity of the implanted tissues could be repeatedly measured according to a standard of neovascularization assessed by microvessel counts and VEGF expression [18].

Based on the model, the implanted AVM tissues showed the highest angiogenesis compared to other cerebrovascular disorders, cavernous malformation, and venous angioma, indicating that the AVM niduses were more likely to be active and progressive. The implanted AVM tissues previously treated with embolization exhibited the highest angiogenic activity, followed by untreated and gamma knife treated AVM tissues; this might explain why AVM recurrence after intravascular embolization was more common. Moreover, this rat cornea model containing human AVM tissues could be used for evaluating molecular mechanisms of the neovascularization process over time [66].

### 3.8. The AVM Lesions by Gene Manipulation

Hereditary hemorrhagic telangiectasia (HHT) is an autosomal dominant vascular disorder characterized by recurrent nosebleeds, mucocutaneous telangiectases, and AVM formations in the brain and other visceral organs [67]. Heterozygous mutations in two genes, endoglin (*Eng*) and Activin receptor-like kinase 1 (*Alk1*), respectively, cause HHT type 1 and type 2. It is logical that animal models containing spontaneous or induced AVM lesions could be generated by regulating the genes.

Knockdown of* Alk1* by its splice-site blocking morpholino caused a spectrum of morphologic and functional defects as AVM lesions in zebrafish embryos [68]. The transgenic mice lacking both alleles of either* Eng* or* Alk1* genes died in embryonic period due to defects in vessel and heart developments [19, 21, 69]. Both* Eng* ^+/−^ and* Alk1* ^+/−^ haploinsufficient mice could be successfully generated. These mice develop vascular lesions in various organs, but spontaneous lesions in the brain were modest in* Eng* ^+/−^ mice and minimal in* Alk1* ^+/−^ mice [20, 22]. A research group headed by Su et al. induced cerebral microvascular dysplasia by transferring virus-mediated VEGF gene to the brain of* Eng* ^+/−^ or* Alk1* ^+/−^ adult mice [23–25]. The AVM-like capillary dysplasia was more pronounced in* Eng* ^+/−^ mice than in* Alk1* ^+/−^ mice. Interestingly, increased cerebral perfusion by intraventricular infusion of hydralazine or nicardipine after VEGF delivery promoted capillary dysplasia in* Alk1* ^+/−^ mice. These studies demonstrated that VEGF delivery into the brain of wide type mice led to increased microvessel counts but not microvascular dysplasia, and saline injection did not cause significant microvascular changes even in the haploinsufficient mice, approving that the development and progression of AVM lesions in adult brains were possible, when hereditary variation was combined with endogenous or exogenous growth factor delivery. Although sharing the somewhat alike phenotype, the induced local microvascular dysplasias were not enough to stand for direct models of the disease. However, they might be useful in identifying the possible factors which took a role in the pathogenesis of AVMs.

The conditional knockout technique with Cre/LoxP recombination system made it possible to delete target genes at the planned time or in the expected cells, because the Cre enzyme expression could be precisely controlled. Conditional deletion of both* Alk1* alleles in adult mice by tamoxifen-inducible Cre resulted in AV fistula formations and spontaneous hemorrhage mostly in the lung, gastrointestinal track, and uterus, but not remarkably in the brain, although de novo vascular malformation lesions developed upon induction of skin wounding in these mice [26]. The similar phenomenon could be observed in conditional* Eng* deletion mice, in which vessel abnormalities mimicking human AVM nidus were induced in the brain with the presence of angiogenic stimulation such as mechanical injury or VEGF delivery [27]. Meanwhile, Su's research group successfully produced AVM lesions in the adult mouse brain resembling the human disease, by injecting vectors expressing both Cre and VEGF into the basal ganglia of Alk1^2LoxP/2LoxP^ and *Eng* ^2LoxP/2LoxP^ mice [28, 29]. The results showed that cerebrovascular lesions were more severe in Alk1^2LoxP/2LoxP^ mice due to more effective gene deletion. In fact, regional deletion of* Eng* caused more severe cerebrovascular malformation per copy than* Alk1* with VEGF stimulation. These models were promising for evaluating the pathogenic mechanisms of AVMs and for discovering potential medical therapies to slow AVM growth and stabilize the rupture-prone abnormal vasculature.

Antenatal deletion of both* Alk1* alleles in restricted endothelial cells (ECs) caused severe and fatal visceral arteriovenous malformations [70]. Conditional deletion of* Alk1* specifically in ECs in adult mice resulted in AVM formations in the intestine, lung, and around ear-tag wounds, as well as in the brain area previously injected with vectors expressing EVGF [30]. Model mice died in 6–13 days due to bleeding and anemia. This phenotype was the same as that of mice with global* Alk1* deletion [26], indicating the pivotal role of ECs in pathogenesis of AVMs. In contrast, deletion of* Alk1* in pericytes alone was not sufficient to initiate AVM development in adult mice. Similarly, endothelial specific deletion of* Eng* led to endothelial proliferation and AVM formations in neonatal retina and local venomegaly in the adult skin induced by mechanical and VEGF stimulation [31]. Owing to the lack of brain-dominant lesions, Milton et al. successfully generated mouse models with spontaneous AVMs in the brain and/or spinal cord by deleting* Alk1* in the embryo by SM22-Cre, which was expressed in smooth muscle cells, ECs, and some other cell types in different organs [32]. Most of the mice showed a paralysis or lethality phenotype due to internal hemorrhage during the first 10 to 15 weeks of life. However, the mice that survived this period showed reduced lethality rates even though they carried multiple AVMs. Choi et al. created a similar model with the spontaneous onset AVMs in *Eng* ^2LoxP/2LoxP^; with SM22-Cre expressed mice, in which AVMs were found in the central nervous system and intestine, more than half of the mice died from internal hemorrhage before 6 weeks of age [27]. These distinctive models possibly allowed us to study pathophysiology of AVM rupture.

Other genes involved in angiogenesis would also be manipulated to create AVM models. Taking essential roles in vascular development and remolding, Notch signaling pathway was upregulated in human AVMs and might be an important molecular regulator of AVM pathogenesis [71]. Both Notch loss-of-function and gain-of-function mutations impair vascular development, resulting in arteriovenous shunting in zebrafish and mouse embryos, indicating that proper spatial and temporal patterns of Notch activity were critical for angiogenesis [33, 72, 73]. Postnatal overexpression of constitutively active* Notch4* in the endothelium by the tetracycline-regulatory system elicited cerebral arteriovenous shunting in mice, and gene repression reversed the AVM progression [33]. Further analysis of this model showed that AVMs arose from enlargement of preexisting microvessels in size of capillaries, without smooth muscle cell coverage but with high blood flow, implying cellular and hemodynamic mechanisms underlying AVM pathogenesis [74]. Similarly, endothelial expression of constitutively active* Notch1* led to AVM formations in the neonatal mouse brain, and activation of Notch1 in adult mouse caused AVM formations in other organs, but not in the brain [73, 74].

The lack of matrix Gla protein (Mgp) also caused AVMs in mice. Cerebral enlarged vessels and direct connections between arteries and veins were detected in the* Mgp* ^−/−^ mice, but not in* Mgp* ^+/−^ mice at 4 weeks of age. Mgp is a bone morphogenetic protein (BMP) inhibitor. Increased BMP activity due to the deficiency of Mgp induced expression of Alk1 and subsequently enhanced expression of Notch ligands Jagged 1 and Jagged 2, which abnormally increased Notch activity. As expected, reduced Jagged expression in the* Mgp* ^−/−^ mice by crossing them with Jagged deficient mice normalized endothelial differentiation and prevented AVM formations [34]. Moreover, deletion of endothelial* Rbpj*, a mediator of Notch signaling, in postnatal day one resulted in features of AVMs in the mouse brain, including abnormal AV shunting and tortuous vessels. Deletion of the* Rbpj* gene in adult mice did not cause brain AVMs [35].

Cerebrovascular abnormalities, AVM formations, and hemorrhage occurred spontaneously in some cases where relevant genes were directly or conditionally deleted at the antenatal or postnatal stages, although in most cases, the model mice either displayed minimal vascular lesions or obvious vascular lesions out of the brain. The spontaneous cerebral AVM lesions partially simulated the natural clinical course of the disease, but the lesions lacked uniformity and reproducibility in size and location. Focal angiogenic stimulation based on gene deficiency helped to create adult onset models of induced AVM lesions in the brain. These models containing comparable AVM lesions might be more suitable for mechanism and therapeutic studies. In spite of posing disadvantages such as complicated procedures, high expanding, and being time consuming, the models by gene manipulation were unique for investigating the AVM pathogenesis and testing new therapies.

## 4. Discussion and Conclusions

As shown in Tables 1 and 2, animal models in studying AVMs were diverse. In the early period, investigators produced hypoperfusion and/or venous hypertension in the whole or regional brain by extra- or intracranial arteriovenous fistulae, to evaluate the hemodynamic and pathophysiological changes of AVM adjacent parenchyma in the presence of an AVM lesion or after its resection, so as to explain the symptoms and to protect against postoperative complications. The discovery of the special vascular structures as the AVM nidus in animals (the RM in artiodactyls and the venous plexus in rats) made it possible to practice the occlusive treatments (endovascular embolization and radiotherapy) and to analyze therapeutic effects. Lately, the manipulation of angiogenesis-related genes helped to create mutant mice with real AVM lesions in the brain. With the improvement of its stability, this promising model was worthwhile for studying the mechanisms about the origination, progression, and rupture of AVMs. Other ingeniously designed models, including induced AVM-like lesions in the dog brain and implanted transgenic arteriovenous fistula from mice to rats, possessed their own values to investigate pathogenesis and novel treatments. The rat cornea model was to evaluated angiogenic mechanisms especially of human AVM specimens.

An ideal AVM model, which completely shared the same anatomic, physiologic, biological, and clinical features as human AVM disease, was lacking. Even the transgenic mice model carried out with spontaneous but systematic vascular malformation lesions could not fully represent the sporadic cases mostly seen in clinic. In spite of limitations, these various models provided assistance to answer particular questions in the study of AVMs.

The origin of AVM is still a mystery. It was generally believed that the vascular disorder was initiated during embryonic development. However, evidences from animal models demonstrated that postnatal formations of AVMs were possible, due to the two causal factors of angiogenic stimulation and gene deficiency. With genome-wide association study, investigators attempted to identify mutant genes associated with AVM susceptibility in sporadic AVM patients. The possible involved genes included Alk1, Eng, interleukin-6 (IL-6), and interleukin-1*β* (IL-1*β*) with single nucleotide polymorphisms (SNPs), but the limited results were inconsistent [75, 76].

The mechanisms that underlie AVM growth and progression remain poorly understood. Abnormally high blood flow and shear forces in nidal vessels activated molecular pathways in smooth muscle cells and ECs. Hypoperfusion and hypoxia in the nidal and surrounding tissues stimulated angiogenesis and inflammatory reactions. Both of them lead to vascular proliferation and remodeling [77]. These hypothetic mechanisms were demonstrated in Yassari's and Pietila's animal models and were also supported from the analysis of resected human AVM specimens, where the related factors like transforming growth factor (TGF), VEGF, matrix metalloproteinase-9 (MMP-9), BMP, cellular adhesion molecules, and so on were overexpressed [78].

Intracranial hemorrhage is the most severe and most common clinical presentation of AVM patients. Risk factors associated with AVM rupture include certain genetic mutations, intranidal aneurysms, exclusive or restricted venous drainage, deep or infratentorial location, and history of previous hemorrhage [79–81]. SNPs of IL-6, tumor necrosis factor-*α* (TNF-*α*), MMP-9, and other genes in AVM specimens appeared to influence clinical course of AVM rupture [82]. However, the exact molecular mechanisms of AVM rupture need to be scrutinized. Studies of human AVM lesions indicated that multiple mechanisms including inflammation, extracellular matrix remodeling, ECs abnormalities, and immature nidal vessels all likely contributed to hemorrhagic tendency [83]. Further researches are anticipated by using animal models with spontaneous hemorrhagic AVM lesions.

Among the conventional treatments, microsurgical resection is currently recommended for Spetzler-Martin Grades I and II AVMs. For high-grade AVMs, combined treatments are often used lacking a standard procedure. Given that the majority of high-grade lesions cannot be treated without relatively high morbidity and mortality, new biological therapies and gene therapies are under development aiming toward vascular remodeling. A study showed that losartan, an angiotensin II receptor antagonist, attenuated abnormal blood vessel morphology in the* Alk1* knockout zebrafish through modulating the BMP signaling pathway [84]. In the Alk1^2LoxP/2LoxP^ mice model with focal AVMs by virus-mediated Cre and VEGF, the induced angiogenesis and vascular dysplasia were attenuated by administration of VEGF antagonist bevacizumab [85], which later successfully treated a female HHT patient [86]. Moreover, with the deeper understanding the therapeutic mechanisms of radiosurgery in Yassari's and Lawton's models, vascular targeting therapy might improve the obliterating rate and decrease the complications of radiosurgery.

We hope this review would provide the basic of currently available AVM models. The diverse techniques and methods displayed here might shed light on the creation of preferable AVM models in the future, overall promoting further studies of the disease.

## Figures and Tables

**Table 1:** The highlights of the original models for AVMs.

Type	Author [Reference]	Year	Animal	Characteristics	Applications
Carotid jugular fistula (CJF)	Spetzler et al. [6]	1978	cat	Different types of CJF to cause cerebral hypoperfusion and/or draining venous hypertension, fistula opening and closing simulating the presence and resection of the AVM lesion	To evaluate the hemodynamic and pathophysiological changes of AVM adjacent parenchyma, but not the AVM lesion itself. To explain the AVM symptoms and the postoperative complications
	Morgan et al. [7]	1989	rat		
	Bederson et al. [8]	1991	rat		
	Hai et al. [9]	2002	rat		
	Scott et al. [10]	1978	monkey		

Intracranial arteriovenous fistula	Numazawa et al. [11]	2005	dog	A venous graft shunting blood from a branch of the MCA to the SSS, the arterial territory as the blood stolen tissue surrounding AVMs	As above, more precisely in regional parenchyma, but not in the whole brain

Rete mirabile (RM) as the AVM nidus	Chaloupka et al. [12]	1994	pig	Inserting a needle to communicate the RM with the cavernous sinus	To Test and evaluate the embolization and radiosurgery therapy
	Massoud et al. [13]	1994	pig	Establishing a CJF to retrogradely drain the blood from the RM	
	Qian et al. [14]	1999	sheep		

Venous plexus as the AVM nidus	Yassari et al. [15]	2004	rat	Creating a CJF, arterialized venous vessels as an extracranial AVM lesion	To study molecular mechanism of AVM development and the effect of radiosurgery

AVM-like lesions derived from implants	Pietilä et al. [16]	2000	dog	A pedicled muscle graft implanted to the brain with an arteriovenous bypass	To emonstrate angiogenic mechanism of the AVM formation and development

Xenograft arteriovenous fistula	Lawton et al. [17]	2004	rat	Inserting an arterial graft from transgenic mice between the CCA and the EJV of nude rats	To evaluate the mechanism of radiotherapy and to develop novel therapies

AVM tissue -implanted cornea model	Konya et al. [18]	2005	rat	Transplanting human AVM tissues to the rat's cornea	To evaluate the angiogenic property and its mechanism of human AVM specimens

AVM lesions by gene manipulation	Details in Table 2				

AVM: arteriovenous malformation; MCA: middle cerebral artery; SSS: superior sagittal sinus; CCA: common carotid artery; EJV: external jugular vein.

**Table 2:** The highlights of AVM models by gene manipulation.

Type	Author [Reference]	Year	Animal	Characteristics	Applications
AVM lesions by gene manipulation	Bourdeau et al. [19] Satomi et al. [20]	1999 2003	mouse	Generating *Eng* ^+/−^ mutant mice: modest cerebrovascular abnormality	To investigate the pathogenic mechanisms of AVMs in genetic factors
	Oh et al. [21] Srinivasan et al. [22]	2000 2003	mouse	Generating *Alk1* ^+/−^ mutant mice: minimal cerebrovascular abnormality	
	Xu et al. [23]	2004	mouse	Focal virus-mediated VEGF gene transferred in the brain of *Eng* ^+/−^ mice: cerebral microvascular dysplasia	To investigate the pathogenic mechanisms of AVMs in genetic and environmental factors
	Hao et al. [24, 25]	2008	mouse	Focal virus-mediated VEGF gene transferred in the brain of *Alk1* ^+/−^ mice: cerebral microvascular dysplasia, less severe compared to *Eng* ^+/−^ mice, promoted by increased cerebral perfusion	
	Sung et al. [26]	2009	mouse	Conditional knockout of *Alk1* ^2LoxP/2LoxP^ by globally expressed Cre in adult mice: AV fistula formations and spontaneous hemorrhage in other organs, not remarkable in the brain	To investigate the pathogenic and hemorrhagic mechanisms of AVMs
	Choi et al. [27]	2014	mouse	Conditional knockout of *Eng* ^2LoxP/2LoxP^ by globally expressed Cre in adult mice: no remarkable effects on brain vasculature, angiogenesis and cerebrovascular lesions mimicking human AVM nidus developed with focal virus-mediated VEGF gene transferred	
	Walker et al. [28]	2011	mouse	Focal virus-mediated Cre and VEGF gene transferred in *Alk1* ^2LoxP/2LoxP^ mice: AVM lesions resembling the human disease	To investigate the pathogenic mechanisms of AVMs and to test the potential treatments
	Choi et al. [29]	2012	mouse	Focal virus-mediated Cre and VEGF gene transferred in *Eng* ^2LoxP/2LoxP^ mice: AVM lesions resembling the human disease	
	Chen et al. [30]	2014	mouse	Conditional knockout of *Alk1* ^2LoxP/2LoxP^ specifically in endothelial cells in adult mice: AVM formation and spontaneous hemorrhage in other organs and brain areas with previously focal virus-mediated VEGF gene transferred	To evaluated the role of endothelia in the pathogenesis of AVMs
	Mahmoud et al. [31]	2010	mouse	Conditional knockout of *Eng* ^2LoxP/2LoxP^ specifically in endothelial cells in postnatal mice: endothelial proliferation and AVM formation in neonatal retina, local venomegaly in adult skin induced by angiogenic stimulation	
	Milton et al. [32]	2012	mouse	Mating *Alk1* ^2LoxP/2LoxP^ mice with SM22-Cre mutant mice: spontaneous AVMs in the brain and spinal cord, partial lesions undergoing spontaneous hemorrhage	To investigate the hemorrhagic mechanisms of AVMs and to test the potential treatments
	Choi et al. [27]	2014	mouse	Mating *Eng* ^2LoxP/2LoxP^ mice with SM22-Cre mutant mice: spontaneous AVMs in the brain and spinal cord, partial lesions undergoing spontaneous hemorrhage	
	Murphy et al. [33]	2008	mouse	Induced overexpression of constitutively active *Notch4* and *Notch1* in neonatal mice: hallmarks of AVMs in the brain and cerebral hemorrhage	To investigate the pathogenic mechanisms of AVMs in genetic factors
	Yao et al. [34]	2013	mouse	Generating *Mgp* ^−/−^ mutant mice: vascular enlargement and AV shunting	
	Nielsen et al. [35]	2014	mouse	Deleting *Rbpj* gene in neonatal mice: AV shunting and tortuous vessels	

AVM: arteriovenous malformation; VEGF: vascular endothelial growth factor.
